# Immunopathogenesis of Progressive Scarring Trachoma: Results of a 4-Year Longitudinal Study in Tanzanian Children

**DOI:** 10.1128/IAI.00629-19

**Published:** 2020-03-23

**Authors:** Tamsyn Derrick, Athumani M. Ramadhani, David Macleod, Patrick Massae, Elias Mafuru, Malisa Aiweda, Kelvin Mbuya, William Makupa, Tara Mtuy, Robin L. Bailey, David C. W. Mabey, Martin J. Holland, Matthew J. Burton

**Affiliations:** a Department of Clinical Research, London School of Hygiene and Tropical Medicine, London, United Kingdom; b Kilimanjaro Christian Medical Centre, Moshi, Tanzania; c Department of Infectious Disease Epidemiology, London School of Hygiene and Tropical Medicine, London, United Kingdom; Yale University School of Medicine

**Keywords:** *Chlamydia trachomatis*, conjunctiva, inflammation, mucosa, scarring, immunopathogenesis

## Abstract

Trachoma is initiated during childhood following repeated conjunctival infection with Chlamydia trachomatis, which causes a chronic inflammatory response in some individuals that leads to scarring and in-turning of the eyelids in later life. There is currently no treatment to halt the progression of scarring trachoma due to an incomplete understanding of disease pathogenesis. A cohort study was performed in northern Tanzania in 616 children aged 6 to 10 years at enrollment. Every 3 months for 4 years, children were examined for clinical signs of trachoma, and conjunctival swabs were collected for C. trachomatis detection and to analyze the expression of 46 immunofibrogenic genes. Data were analyzed in relation to progressive scarring status between baseline and the final time point. Genes that were significantly associated with scarring progression included those encoding proinflammatory chemokines (*CXCL5*, *CCL20*, *CXCL13*, and *CCL18*), cytokines (*IL23A*, *IL19*, and *IL1B*), matrix modifiers (*MMP12* and *SPARCL1*), immune regulators (*IDO1*, *SOCS3*, and *IL10*), and a proinflammatory antimicrobial peptide (*S100A7*). In response to C. trachomatis infection, *IL23A* and *PDGF* were significantly upregulated in scarring progressors relative to in nonprogressors. Our findings highlight the importance of innate proinflammatory signals from the epithelium and implicate interleukin 23A (IL-23A)-responsive cells in driving trachomatous scarring, with potential key mechanistic roles for *PDGFB*, *MMP12*, and *SPARCL1* in orchestrating fibrosis.

## Abstract (precis)

Trachoma is initiated during childhood following repeated conjunctival infection with Chlamydia trachomatis, which causes a chronic inflammatory response in some individuals that leads to scarring and in-turning of the eyelids in later life. There is currently no treatment to halt the progression of scarring trachoma due to an incomplete understanding of disease pathogenesis. A cohort study was performed in northern Tanzania in 616 children aged 6 to 10 years at enrollment. Every 3 months for 4 years, children were examined for clinical signs of trachoma, and conjunctival swabs were collected for C. trachomatis detection and to analyze the expression of 46 immunofibrogenic genes.

## INTRODUCTION

Trachoma is a neglected tropical disease and the leading infectious cause of blindness worldwide. Disease is initiated by repeated infection of the conjunctival epithelium by the intracellular bacterium Chlamydia trachomatis, which occurs during childhood in areas where trachoma is endemic. Ocular C. trachomatis infection stimulates a chronic pathological inflammatory response in a proportion of exposed individuals, which leads to scarring of the conjunctiva. Conjunctival fibrosis tightens the eyelid, drawing inwards the lid margin (entropion) and eyelashes (trichiasis) such that they cause mechanical damage to the cornea. Without intervention (epilation of the lashes or surgery to correct lid orientation) this damage results in pain, secondary infection, and ultimately blindness. In 2017, 165 million people lived in districts requiring public health interventions for trachoma and 231,447 people worldwide were managed for trichiasis (1).

Mass antibiotic distribution with azithromycin is administered for population control of C. trachomatis in districts requiring intervention, alongside facial cleanliness promotion and environmental improvements to reduce transmission. However, scarring disease progresses in previously exposed individuals in the absence of evidence of ongoing C. trachomatis infection, suggesting that service provision for trichiasis management will be required for many years in districts where it was formerly endemic (2, 3). There is currently no treatment to halt the progression of established scarring, partly due to an incomplete understanding of the immunopathophysiological process.

Following pathogen recognition and initiation of inflammation by the conjunctival epithelium, an adaptive immune cell-mediated response involving Th1 cells, classically activated macrophages, and gamma interferon (IFN-γ) is believed to be essential to clear ocular C. trachomatis infection (4–6). NK cells are an additional source of IFN-γ, and there is evidence that Th17 cells and associated cytokines are involved in the antichlamydial immune response (4, 7, 8). Following pathogen clearance, there is an extended period of inflammation and wound healing, which is thought to be characterized by the presence and activity of neutrophils and growth and matrix factors and by a reduction in the expression of mucin genes (4, 8). These data have been gathered almost entirely from cross-sectional studies, and, as a result, the factors driving healthy wound healing versus pathological inflammation and fibrosis have not been differentiated.

We recently reported the results of a cohort study that investigated the association between conjunctival C. trachomatis infection and clinically visible inflammatory episodes with scarring progression in children over a 4-year period (9). The study took place from 2012 to 2016 in a region of northern Tanzania where trachoma is endemic. Scarring progression was observed in 103/448 (23%) individuals and was strongly associated with an increasing proportion of episodes with papillary inflammation (TP) (equivalent to P2 or P3 of the Detailed WHO Trachoma Grading System [2]). There were also marginal associations between “trachomatous inflammation—follicular” (TF) and C. trachomatis with scarring progression, which were shown to be mediated through TP. This suggests that other factors causing individual differences in TP contributed to scarring progression. However, the immune mediators driving TP and their cellular origins are unknown.

The aim of this study was to determine which components of the inflammatory response were most strongly associated with 4-year scarring progression and pathological TP. We further examined whether individuals with scarring progression responded differently at the gene expression level to C. trachomatis infection relative to nonprogressors.

## RESULTS

A detailed description of the longitudinal study design and primary outcomes has been published elsewhere (4, 9, 10). Briefly, of the 666 eligible children, 616 were enrolled in the cohort study. Of these, 448 remained in the primary outcome analysis of factors associated with 4-year scarring progression. Scarring progression was observed in 23% (103/448) of individuals and was associated with increasing proportion of TP episodes. Gene expression was analyzed at study time points 1 to 5, 7, 9, 11, 13, 15, and 17. The number of participants seen at each time point and the number in which C. trachomatis and TP were detected are shown in Table 1. In addition to the endogenous control genes *HPRT1* and *GAPDH*, 46 genes of interest were quantified in all available samples at each of these time points. Three genes (*FGF2*, *SERPINB4-SERPINB3*, and *IL22*) and 61 observations were excluded from all analyses due to >10% missing data.

**TABLE 1:** Number of individuals for which gene expression data were available at each of the study time points, with C. trachomatis infection and TP statuses

Time point	No. of participants sampled	No. (%) with^*a*^:	
		C. trachomatis infection	TP
1	506	78 (15.4)	99 (19.6)
2	536	82 (15.3)	107 (20)
3	466	54 (11.6)	68 (14.6)
4	466	6 (1.3)	10 (2.1)
5	477	12 (2.5)	35 (7.3)
7	472	21 (4.4)	14 (3)
9	450	12 (2.7)	12 (2.7)
11	426	45 (10.6)	12 (2.8)
13	444	33 (7.4)	13 (2.9)
15	380	49 (12.9)	20 (5.3)
17	426	21 (4.9)	14 (3.3)

a The absolute number of participants in which C. trachomatis or TP was detected at each time point is shown; chronic and new infections in the same or different children are not differentiated.

The association between gene expression and scarring progression was analyzed using random effects logistic regression models of the longitudinal data, clustering on participant identification number and adjusting for C. trachomatis infection, age, and sex. Of the 10 genes most strongly associated with 4-year scarring progression, only *SPARCL1* (fold change [FC] = 0.54; *P* = 1.36 × 10^−5^) was downregulated in scarring progressors, whereas *CXCL5*, *SOCS3*, *IL23A*, *IL19*, *CCL20*, *IDO1*, *MMP12*, *S100A7*, and *IL1B* were upregulated (Table 2). All upregulated genes had marginal fold changes of less than 1.4. With the exception of NCAM1, there was strong evidence that expression of all genes was altered with C. trachomatis infection (Table 2). The three most strongly upregulated genes (FC > 5) in response to infection were *IFNG*, *IL21*, and *CCL18*, and the three most strongly downregulated genes (FC < 0.32) were *MUC7*, *MUC5AC*, and *SPARCL1*. There was evidence of an association between 20 genes and sex; 16 of these genes were upregulated in females. The top three genes most strongly associated with female sex were *IL21*, *IL17A*, and *IFNG*. Evidence was found for an association between age and all genes except *ALOX5*, *CD247*, *IL12B*, *VIM*, *PDGFB*, and *TGFB1*. Out of the genes associated with age, the majority (29/37) had negative fold changes, indicating that expression was higher in younger participants.

**TABLE 2:** Associations between gene expression and scarring progression, adjusted for C. trachomatis infection, age, and sex^*a*^

Target	Scarring progression			Adjusted for:								
				Infection			Age			Sex		
	FC	LCI–UCI	*P* value	FC	LCI–UCI	*P* value	FC	LCI–UCI	*P* value	FC	LCI–UCI	*P* value
Arachidonate 5-lipoxygenase (*ALOX5*)	0.96	0.91–1.01	0.111	0.61	0.58–0.64	**2.79E–103**	1.01	1.00–1.02	0.036	1.01	0.97–1.06	0.596
Chemokine ligand 18 (*CCL18*)	1.02	0.87–1.18	0.837	5.09	4.33–5.99	**8.45E–86**	0.87	0.84–0.90	**2.02E–17**	1.11	0.98–1.26	0.108
Chemokine ligand 2 (*CCL2*)	1.06	0.96–1.18	0.223	4.31	3.84–4.85	**3.43E–131**	0.93	0.91–0.95	**3.28E–11**	1.08	0.99–1.17	0.087
Chemokine ligand 20 (*CCL20*)	1.16	1.04–1.28	**0.008**	1.58	1.43–1.76	**1.01E–17**	0.94	0.92–0.96	**5.21E–08**	1.06	0.97–1.16	0.22
CD247 molecule (*CD247*)	0.95	0.90–1.01	0.099	1.92	1.80–2.05	**7.21E–90**	0.99	0.98–1.00	0.063	1.05	1.00–1.10	0.074
CD274 molecule (*CD274*)	1.08	1.00–1.17	0.048	3.14	2.89–3.42	**4.42E–157**	0.92	0.91–0.94	**8.19E–21**	1.13	1.06–1.21	**1.68E–04**
Epithelial cadherin (*CDH1*)	0.98	0.94–1.03	0.476	0.63	0.59–0.67	**2.27E–47**	1.03	1.02–1.04	**2.28E–07**	1.03	0.99–1.07	0.176
Neuronal cadherin (*CDH2*)	0.91	0.81–1.03	0.148	0.59	0.52–0.65	**2.13E–21**	1.11	1.08–1.14	**8.09E–14**	1.11	0.99–1.23	0.063
Connective tissue growth factor (*CTGF*)	0.92	0.84–1.01	0.07	0.78	0.71–0.86	**1.57E–07**	1.06	1.03–1.08	**1.26E–07**	0.9	0.83–0.97	**0.006**
Chemokine ligand 13 (*CXCL13*)	1	0.86–1.16	0.978	4.72	4.03–5.53	**1.05E–82**	0.85	0.82–0.88	**1.21E–21**	1.31	1.15–1.49	**4.16E–05**
Chemokine ligand 5 (*CXCL5*)	1.37	1.13–1.65	**0.001**	1.56	1.36–1.79	**2.98E–10**	0.83	0.80–0.87	**7.10E–19**	0.99	0.84–1.17	0.93
Defensin, beta 4B, defensin, beta 4A (*DEFB4B*-*DEFB4A*)	1.19	0.98–1.44	0.082	1.65	1.44–1.89	**5.82E–13**	0.86	0.83–0.90	**5.44E–12**	1.3	1.10–1.53	**0.002**
Dual oxidase 2 (*DUOX2*)	1.09	0.97–1.23	0.143	1.36	1.24–1.48	**9.63E–12**	0.9	0.88–0.92	**5.68E–17**	1.35	1.22–1.49	**3.32E–09**
Indoleamine 2,3-dioxygenase 1 (*IDO1*)	1.23	1.06–1.44	**0.008**	2.42	2.18–2.69	**2.54E–62**	0.86	0.83–0.89	**1.58E–18**	1.41	1.24–1.61	**2.45E–07**
Interferon gamma (*IFNG*)	1.03	0.93–1.13	0.595	7.75	7.03–8.55	**0.00E+00**	0.95	0.93–0.97	**3.12E–07**	1.29	1.19–1.40	**5.84E–10**
Interleukin 10 (*IL10*)	1	0.93–1.08	0.966	2.6	2.39–2.84	**1.11E–104**	0.94	0.92–0.95	**3.32E–15**	1.12	1.05–1.20	**2.97E–04**
Interleukin 12 beta (*IL12B*)	0.94	0.86–1.04	0.233	3.76	3.40–4.16	**1.81E–147**	1.02	1.00–1.04	0.076	1.08	1.00–1.18	0.052
Interleukin 17A (*IL17A*)	1.12	0.99–1.26	0.074	3.67	3.26–4.13	**2.04E–103**	0.88	0.85–0.90	**1.08E–23**	1.4	1.27–1.55	**1.11E–10**
Interleukin 19 (*IL19*)	1.25	1.07–1.46	**0.004**	3.33	2.92–3.79	**1.35E–72**	0.86	0.83–0.89	**5.96E–20**	1.47	1.29–1.68	**7.11E–09**
Interleukin 1 beta (*IL1B*)	1.14	1.02–1.27	**0.017**	1.96	1.75–2.18	**2.93E–33**	0.91	0.89–0.93	**1.80E–16**	0.99	0.90–1.08	0.79
Interleukin 21 (*IL21*)	1.07	0.94–1.21	0.303	6.49	5.65–7.46	**5.42E–153**	0.88	0.86–0.90	**7.36E–22**	1.42	1.28–1.58	**3.19E–11**
Interleukin 23A (*IL23A*)	1.14	1.05–1.23	**0.002**	1.92	1.76–2.10	**1.77E–48**	0.93	0.91–0.94	**3.04E–17**	1.04	0.97–1.12	0.256
Interleukin 6 (*IL6*)	1.1	0.97–1.24	0.124	1.87	1.66–2.11	**2.41E–24**	0.96	0.94–0.99	**0.005**	0.98	0.88–1.08	0.668
Interleukin 8 (*IL8*)	1.1	1.00–1.20	0.042	1.32	1.21–1.44	**3.19E–10**	0.97	0.95–0.99	**0.004**	0.99	0.92–1.07	0.791
Matrix metallopeptidase 12 (*MMP12*)	1.2	1.05–1.38	**0.008**	3.22	2.88–3.59	**6.72E–95**	0.89	0.86–0.91	**1.45E–15**	1.14	1.02–1.28	**0.024**
Matrix metallopeptidase 7 (*MMP7*)	1	0.85–1.18	0.993	0.51	0.48–0.55	**4.95E–67**	0.96	0.93–0.99	**0.022**	0.92	0.80–1.06	0.257
Matrix metallopeptidase 9 (*MMP9*)	1.08	0.97–1.22	0.173	2.28	2.06–2.52	**5.07E–58**	0.94	0.92–0.97	**2.56E–06**	1	0.90–1.10	0.97
Mucin 1, cell surface associated (*MUC1*)	1.03	0.97–1.09	0.291	0.74	0.70–0.79	**1.04E–23**	0.98	0.97–0.99	**2.93E–04**	1.08	1.03–1.13	**0.002**
Mucin 4, cell surface associated (*MUC4*)	1.08	1.00–1.16	0.042	0.73	0.68–0.78	**6.13E–21**	0.96	0.95–0.98	**1.05E–05**	1.03	0.97–1.10	0.306
Mucin 5AC, oligomeric mucus/gel-forming (*MUC5AC*)	0.89	0.75–1.05	0.181	0.27	0.24–0.30	**3.42E–96**	1.15	1.11–1.20	**5.16E–15**	1.04	0.91–1.20	0.549
Mucin 7, secreted (*MUC7*)	0.99	0.83–1.19	0.955	0.31	0.27–0.34	**1.47E–94**	1.05	1.01–1.09	**0.011**	0.66	0.57–0.77	**7.37E–08**
Marginal zone B and B1 cell-specific protein (*MZB1*)	1.07	0.91–1.26	0.402	3.09	2.73–3.49	**1.43E–72**	0.91	0.88–0.94	**8.00E–08**	1.22	1.07–1.40	**0.004**
Neural cell adhesion molecule 1 (*NCAM1*)	0.95	0.87–1.03	0.197	0.95	0.87–1.04	0.29	1.06	1.04–1.08	**4.73E–10**	0.91	0.85–0.98	**0.012**
Natural cytotoxicity triggering receptor 1 (*NCR1*)	1	0.92–1.08	0.978	2.05	1.91–2.21	**3.16E–85**	0.96	0.95–0.98	**1.25E–05**	1.09	1.02–1.17	**0.011**
Platelet-derived growth factor beta polypeptide (*PDGFB*)	0.96	0.91–1.02	0.216	1.39	1.31–1.47	**4.33E–26**	0.99	0.98–1.01	0.411	1.12	1.07–1.18	**1.18E–05**
Prostaglandin-endoperoxide synthase 2 (*PTGS2*)	1.08	0.97–1.19	0.147	1.26	1.14–1.39	**6.72E–06**	0.94	0.92–0.96	**1.39E–07**	0.99	0.91–1.08	0.791
S100 calcium binding protein A4 (*S100A4*)	0.96	0.90–1.02	0.171	0.32	0.30–0.34	**3.52E–227**	1.06	1.05–1.08	**9.30E–19**	0.94	0.89–0.99	**0.014**
Psoriasin-1 (*S100A7*)	1.3	1.05–1.61	**0.015**	4.48	3.74–5.36	**1.14E–59**	0.8	0.77–0.84	**5.62E–21**	1.57	1.31–1.88	**1.21E–06**
Suppressor of cytokine signalling 1 (*SOCS1*)	1.03	0.97–1.10	0.262	1.98	1.84–2.13	**2.03E–73**	0.96	0.95–0.97	**4.58E–10**	1.01	0.96–1.06	0.756
Suppressor of cytokine signalling 3 (*SOCS3*)	1.16	1.06–1.27	**0.002**	1.54	1.40–1.69	**1.78E–18**	0.91	0.89–0.93	**6.65E–20**	1	0.92–1.08	0.996
SPARC-like 1 (hevin) (*SPARCL1*)	0.54	0.41–0.71	**1.36E–05**	0.11	0.09–0.13	**6.32E–103**	1.32	1.24–1.40	**8.14E–20**	0.87	0.69–1.10	0.236
Transforming growth factor, beta 1 (*TGFβ1*)	0.99	0.95–1.03	0.489	1.1	1.05–1.16	**2.22E–04**	1	0.99–1.01	0.578	1.02	0.99–1.06	0.212
Vimentin (*VIM*)	1	0.95–1.04	0.882	1.56	1.47–1.64	**4.26E–56**	0.99	0.98–1.00	0.233	1.03	0.99–1.07	0.157

a Random effects multivariable linear regression models were constructed for each gene in turn. Using the Benjamini and Hochberg method to control for a false discovery rate of <0.05%, *P* values of <0.024 are considered statistically significant (indicated in bold). A fold change of ≥1 in the righthand column shows that the gene was upregulated in females relative to males. FC, fold change; LCI–UCI, lower to upper confidence limits.

To further examine the genes most strongly associated with scarring progression, random effects lasso regression was performed in the longitudinal data set, clustering on participant identifier (ID). After filtering out incomplete cases, 3,622 observations remained in the analysis. In addition to genes associated with sex and age, the final iteration of the model retained 11 genes deemed to be most strongly associated with scarring progression, namely, *SPARCL1*, *CXCL13*, *CCL18*, *CCL20*, *IL10*, *MMP12*, *IDO1*, *IL23A*, *S100A7*, *CXCL5*, and *IL19*. All of these targets were also identified as strongly associated with scarring progression in the linear regression models (Table 2), whereas *CXCL13*, *CCL18*, and *IL10* were additionally identified by the lasso regression only.

Prior analysis of the cohort data set indicated that the marginal association between C. trachomatis infection and 4-year scarring progression was mediated through TP, suggesting that an individual’s response to infection—rather than simply the presence or absence of infection alone—determines their risk of scarring sequelae (9). In order to investigate this further, random effects regression models were performed for each gene in turn; these included an interaction term between scarring progression status and C. trachomatis infection in order to determine whether scarring progressors responded differently to infection relative to nonprogressors. Two genes, *PDGFB* and *IL23A*, showed some evidence of being upregulated in response to C. trachomatis infection by a greater amount in progressors than in nonprogressors (Table 3). In scarring progressors *PDGFB* was upregulated 1.58-fold in response to infection, whereas it was upregulated 1.31-fold in nonprogressors. Similarly, *IL23A* was upregulated 2.33-fold in response to infection in scarring progressors and 1.77-fold in nonprogressors.

**TABLE 3:** Gene expression responses to C. trachomatis infection in scarring progressors relative to those in nonprogressors^*a*^

Target	Scarring progression			Adjusted for:											
				Infection			Interaction (progression × infection)			Age			Sex		
	FC	LCI–UCI	*P* value	FC	LCI–UCI	*P* value	FC	LCI–UCI	*P* value	FC	LCI–UCI	*P* value	FC	LCI–UCI	*P* value
ALOX5	0.97	0.92–1.02	0.206	0.62	0.59–0.66	**8.04E–66**	0.92	0.84–1.02	0.110	1.01	1.00–1.02	0.037	1.01	0.97–1.06	0.607
CCL18	1.00	0.86–1.17	0.979	4.89	4.02–5.94	**2.42E–57**	1.14	0.80–1.63	0.455	0.87	0.84–0.90	**2.07E–17**	1.11	0.98–1.26	0.106
CCL2	1.06	0.96–1.18	0.266	4.27	3.71–4.91	**1.86E–90**	1.04	0.80–1.34	0.780	0.93	0.91–0.95	**3.38E–11**	1.08	0.99–1.17	0.086
CCL20	1.15	1.04–1.29	**0.009**	1.58	1.39–1.79	**1.08E–12**	1.01	0.80–1.26	0.954	0.94	0.92–0.96	**5.24E–08**	1.06	0.97–1.16	0.220
CD247	0.95	0.89–1.01	0.084	1.90	1.76–2.05	**6.10E–61**	1.04	0.90–1.19	0.591	0.99	0.98–1.00	0.064	1.05	1.00–1.10	0.073
CD274	1.08	1.00–1.17	0.050	3.15	2.85–3.49	**1.10E–110**	0.99	0.82–1.18	0.879	0.92	0.91–0.94	**8.11E–21**	1.13	1.06–1.21	**1.692E–04**
CDH1	0.99	0.94–1.04	0.615	0.64	0.59–0.69	**1.50E–31**	0.96	0.83–1.09	0.507	1.03	1.02–1.04	**2.39E–07**	1.03	0.99–1.07	0.180
CDH2	0.91	0.80–1.03	0.128	0.57	0.50–0.66	**2.19E–16**	1.07	0.84–1.35	0.605	1.11	1.08–1.14	**7.73E–14**	1.11	0.99–1.23	0.063
CTGF	0.91	0.83–1.00	0.062	0.77	0.69–0.86	**3.93E–06**	1.05	0.86–1.28	0.664	1.06	1.03–1.08	**1.21E–07**	0.90	0.83–0.97	**0.006**
CXCL13	0.98	0.84–1.15	0.810	4.49	3.71–5.43	**1.76E–54**	1.18	0.84–1.67	0.338	0.85	0.82–0.88	**1.37E–21**	1.31	1.15–1.50	**3.976E–05**
CXCL5	1.34	1.10–1.62	**0.003**	1.47	1.24–1.73	**5.66E–06**	1.22	0.91–1.65	0.189	0.83	0.80–0.87	**8.41E–19**	0.99	0.85–1.17	0.938
DEFB4B-DEFB4A	1.18	0.97–1.44	0.099	1.62	1.37–1.90	**8.04E–09**	1.07	0.80–1.44	0.651	0.86	0.83–0.90	**5.61E–12**	1.30	1.10–1.53	**0.002**
DUOX2	1.10	0.98–1.24	0.110	1.40	1.26–1.55	**5.38E–10**	0.91	0.75–1.10	0.345	0.90	0.88–0.92	**5.29E–17**	1.35	1.22–1.49	**3.489E–09**
IDO1	1.26	1.08–1.47	**0.004**	2.56	2.26–2.90	**2.26E–49**	0.84	0.67–1.05	0.120	0.86	0.83–0.89	**1.32E–18**	1.41	1.24–1.61	**2.547E–07**
IFNG	1.03	0.93–1.13	0.579	7.79	6.93–8.77	**1.42E–257**	0.98	0.79–1.21	0.868	0.95	0.93–0.97	**3.10E–07**	1.29	1.19–1.40	**5.945E–10**
IL10	1.00	0.93–1.08	0.921	2.62	2.36–2.91	**2.15E–74**	0.98	0.81–1.18	0.815	0.94	0.92–0.95	**3.27E–15**	1.12	1.05–1.20	**3.013E–04**
IL12B	0.93	0.84–1.03	0.158	3.62	3.21–4.09	**1.31E–97**	1.13	0.91–1.41	0.270	1.02	1.00–1.04	0.074	1.08	1.00–1.18	0.050
IL17A	1.13	1.00–1.28	0.053	3.80	3.30–4.38	**2.14E–76**	0.89	0.69–1.15	0.384	0.88	0.85–0.90	**9.53E–24**	1.40	1.26–1.55	**1.173E–10**
IL19	1.24	1.06–1.45	**0.008**	3.21	2.74–3.75	**3.88E–48**	1.13	0.85–1.50	0.400	0.86	0.83–0.89	**6.51E–20**	1.47	1.29–1.68	**6.811E–09**
IL1B	1.13	1.02–1.26	**0.025**	1.93	1.69–2.20	**1.14E–22**	1.05	0.83–1.33	0.684	0.91	0.89–0.93	**1.92E–16**	0.99	0.90–1.08	0.794
IL21	1.07	0.95–1.22	0.272	6.61	5.60–7.81	**1.82E–109**	0.94	0.69–1.27	0.686	0.88	0.86–0.90	**6.97E–22**	1.42	1.28–1.58	**3.310E–11**
IL23A	1.11	1.02–1.20	**0.019**	1.78	1.60–1.97	**9.19E–27**	1.30	1.08–1.58	**0.006**	0.93	0.91–0.94	**4.09E–17**	1.04	0.97–1.12	0.242
IL6	1.08	0.95–1.22	0.220	1.78	1.54–2.05	**6.04E–15**	1.18	0.91–1.53	0.210	0.96	0.94–0.99	**0.005**	0.98	0.88–1.08	0.679
IL8	1.09	0.99–1.19	0.067	1.29	1.16–1.43	**1.33E–06**	1.07	0.89–1.29	0.455	0.97	0.95–0.99	**0.004**	0.99	0.92–1.07	0.798
MMP12	1.21	1.05–1.39	**0.008**	3.25	2.85–3.71	**6.62E–68**	0.96	0.76–1.23	0.770	0.89	0.86–0.91	**1.41E–15**	1.14	1.02–1.28	**0.024**
MMP7	1.00	0.85–1.18	0.980	0.52	0.47–0.56	**8.61E–47**	0.99	0.84–1.16	0.873	0.96	0.93–0.99	**0.022**	0.92	0.80–1.06	0.257
MMP9	1.07	0.95–1.21	0.262	2.19	1.94–2.47	**2.36E–37**	1.14	0.91–1.41	0.251	0.94	0.92–0.97	**2.73E–06**	1.00	0.90–1.10	0.979
MUC1	1.04	0.98–1.10	0.189	0.76	0.71–0.82	**1.60E–14**	0.92	0.81–1.05	0.208	0.98	0.97–0.99	**2.72E–04**	1.08	1.03–1.13	**0.002**
MUC4	1.08	1.00–1.17	0.046	0.73	0.67–0.79	**5.21E–15**	1.00	0.86–1.15	0.982	0.96	0.95–0.98	**1.05E–05**	1.03	0.97–1.10	0.306
MUC5AC	0.88	0.75–1.05	0.155	0.26	0.22–0.30	**3.03E–70**	1.09	0.83–1.43	0.534	1.15	1.11–1.20	**4.85E–15**	1.04	0.91–1.20	0.545
MUC7	1.00	0.84–1.20	0.998	0.31	0.27–0.36	**7.59E–65**	0.95	0.74–1.21	0.682	1.05	1.01–1.09	**0.011**	0.66	0.57–0.77	**7.219E–08**
MZB1	1.06	0.90–1.25	0.459	3.02	2.61–3.50	**2.44E–49**	1.07	0.82–1.40	0.616	0.91	0.88–0.94	**8.27E–08**	1.22	1.07–1.40	**0.004**
NCAM1	0.94	0.87–1.03	0.191	0.95	0.85–1.05	0.308	1.02	0.85–1.23	0.815	1.06	1.04–1.08	**4.66E–10**	0.91	0.85–0.98	**0.012**
NCR1	1.00	0.92–1.09	0.957	2.08	1.90–2.26	**1.49E–61**	0.97	0.83–1.13	0.680	0.96	0.95–0.98	**1.23E–05**	1.09	1.02–1.17	**0.011**
PDGFB	0.94	0.89–1.00	0.069	1.31	1.22–1.41	**3.06E–13**	1.20	1.06–1.37	**0.005**	0.99	0.98–1.01	0.427	1.12	1.07–1.18	**9.458E–06**
PTGS2	1.06	0.96–1.18	0.254	1.20	1.07–1.35	**0.002**	1.15	0.93–1.43	0.195	0.94	0.92–0.96	**1.53E–07**	0.99	0.91–1.08	0.803
S100A4	0.97	0.91–1.04	0.401	0.33	0.31–0.36	**6.61E–146**	0.85	0.73–0.99	0.041	1.06	1.05–1.08	**1.14E–18**	0.94	0.89–0.99	**0.013**
S100A7	1.27	1.02–1.58	0.030	4.17	3.36–5.18	**2.16E–38**	1.26	0.85–1.87	0.244	0.80	0.77–0.84	**6.29E–21**	1.57	1.31–1.88	**1.146E–06**
SOCS1	1.03	0.97–1.10	0.301	1.97	1.80–2.15	**6.28E–51**	1.01	0.87–1.19	0.855	0.96	0.95–0.97	**4.65E–10**	1.01	0.96–1.06	0.754
SOCS3	1.16	1.05–1.27	**0.003**	1.53	1.37–1.72	**2.67E–13**	1.00	0.81–1.23	0.992	0.91	0.89–0.93	**6.70E–20**	1.00	0.92–1.08	0.996
SPARCL1	0.54	0.41–0.72	**2.168E–05**	0.11	0.09–0.14	**8.75E–71**	0.94	0.61–1.46	0.787	1.32	1.24–1.40	**8.52E–20**	0.87	0.69–1.10	0.235
TGFB1	0.99	0.94–1.03	0.513	1.10	1.04–1.17	**0.002**	1.00	0.89–1.11	0.959	1.00	0.99–1.01	0.577	1.02	0.99–1.06	0.213
VIM	0.99	0.95–1.04	0.722	1.53	1.44–1.64	**5.89E–37**	1.05	0.93–1.18	0.406	0.99	0.98–1.00	0.237	1.03	0.99–1.07	0.153

a Random effects logistic regression models were constructed for each gene in turn and included an interaction term between scarring progression status and C. trachomatis infection and adjusting for age and sex. Using the Benjamini-Hochberg method to control for a false-discovery rate of <0.05%, *P* values of <0.0254 are considered statistically significant and are indicated in bold. FC, fold change; LCI–UCI, lower to upper confidence limits.

TP was identified as the major risk factor for scarring progression in our previous analysis (9); therefore, the analyses described above were repeated using TP as the primary outcome. In random effects linear regression models, all genes, with the exceptions of *TGFB1* and *MMP7*, had evidence of an association with TP (Table S1); the three most strongly upregulated genes were *CXCL13*, *CCL18*, and *S100A7*, and the three most downregulated genes were *SPARCL1*, *MUC7*, and *MUC5AC*. Lasso regression was performed to identify the genes most strongly associated with TP (Table S2). The final iteration of the model only excluded three genes not deemed to be associated with TP, namely, *MMP7*, *MUC1*, and *NCAM1*. Age and C. trachomatis infection status but not sex were included in the final model. The genes whose expression was most strongly associated with TP were *MZB1*, *MMP9*, *CXCL5*, *PDGFB*, *TGFB1*, *IL17A*, *S100A4*, *IL8*, *S100A7*, *SPARCL1*, and *IDO1*. In order to investigate whether individuals prone to TP (TP was detected at any time point) responded differently to C. trachomatis infection than individuals not prone to TP (in whom TP was never detected throughout the study duration), random effects linear regression models were repeated and included an interaction term between infection and whether any TP was detected. In individuals prone to TP, evidence was found that the expression of *PDGFB*, *S100A7*, *IL23A*, *IL8*, *MMP9*, *CCL2*, *IL19*, *CCL18*, and *IL6* was upregulated, and the expression of *MUC7*, *IDO1*, *S100A4*, and *MMP7* was downregulated in response to C. trachomatis infection, relative to individuals in which TP was never detected (Table S3A). Of these 13 genes, only *S100A7* had a fold change greater than ±1.5. Stratum-specific fold changes are shown in Table S3B.

## DISCUSSION

**FIG 1:**
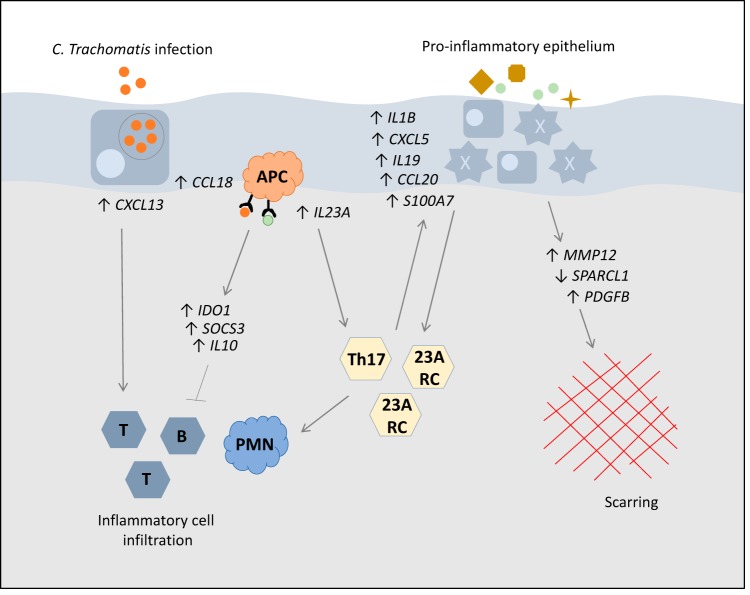
Graphical summary of the genes associated with progressive scarring trachoma and hypothesized molecular pathways of pathogenesis. In response to C. trachomatis infection, scarring progressors had higher expression of *IL23A* and *PDGFB* than nonprogressors, suggesting a bias toward Th17 or other IL-23A-responsive cells (23A RC) and increased fibrosis. The association of increased proinflammatory epithelial (*CXCL5*, *CCL20*, *CXCL13*, *IL19*, *IL1B*, and *S100A7)* and dendritic cell-derived signals *(CCL18*, *IL23A*, and *IL19)* in scarring trachoma is indicative of ongoing epithelial inflammation and continued bias toward IL-23A-responsive cell types, which could form proinflammatory feedback cycles. These responses could be aggravated by external stimuli. Upregulation of the inflammatory regulators *IL10*, *IDO1*, and *SOCS3* supports the presence of chronic or uncontrolled inflammation. Matrix factors *MMP12*, *SPARCL1*, and *PDGFB* may be acting as direct mediators of fibrosis.

In this study, we measured the expression of 46 immune response genes at 11 time points over a 4-year period, analyzing gene expression in relation to 4-year scarring progression status and pathological TP. The genes found to be associated with scarring progression included those encoding proinflammatory chemokines (*CXCL5*, *CCL20*, *CXCL13*, and *CCL18*), cytokines (*IL23A*, *IL19*, and *IL1B*), matrix modifiers (*MMP12* and *SPARCL1*), immune regulators (*IDO1*, *SOCS3*, and *IL10*), and a proinflammatory antimicrobial peptide (*S100A7*). All genes except *SPARCL1* were upregulated. A summary of the putative functions and interactions of these immune mediators is shown in Fig. 1.

*SPARCL1* had the greatest fold change and was the gene most strongly associated with scarring progression in this study. It was also strongly associated with TP and C. trachomatis infection. The downregulation of *SPARCL1* has previously been reported to be associated with trachomatous scarring and inflammation (2, 4, 11). SPARCL1 is a nonstructural secreted protein that regulates the interaction between cells and the extracellular matrix (ECM). Its downregulation has been associated with cell proliferation and metastases in several cancers (12, 13). Knockout of *SPARCL1* in a murine corneal injury model led to the accumulation of inflammatory infiltrates, neovascularization and irregular ECM deposition at the site of injury (14). In contrast to wild-type mice, in which matrix metallopeptidase (MMP) activity was increased shortly after injury and then stabilized, MMP activity (detected through collagenase assay) was significantly greater in knockout mice, and activity increased throughout the duration of the experiment. This increased MMP activity was attributed to the excessive production of irregular collagen (14). MMP12, which is produced by monocytes, ocular epithelial cells, and fibroblasts following injury, was also significantly upregulated in scarring progressors in this study, and *MMP9* was strongly associated with TP. In another murine corneal injury model, MMP12 was found to enhance early wound repair through increasing neutrophil infiltration and epithelial cell migration, suggesting that overexpression in trachoma might contribute to leukocyte infiltration (15). Histological analysis of conjunctival tissue from trichiasis patients has revealed inflammatory cell infiltrates and disrupted collagen structure consistent with the *SPARCL1* knockout corneal injury model (14, 16, 17).

The chemokines CXCL5, CCL20, CXCL13, and CCL18, derived from epithelial and antigen-presenting cells on exposure to microbial stimuli and proinflammatory cytokines, recruit lymphocytes and neutrophils. IL-1β and S100A7 are innate proinflammatory mediators that are upregulated in the epithelium upon microbial exposure; S100A7 has antimicrobial and chemotactic properties (18). *CXCL5*, *CCL18*, *IL1B*, and *S100A7* have consistently been found to be associated with trachomatous inflammation and scarring (2, 8, 11, 19–22). The upregulation of these chemokines and proinflammatory mediators is indicative of ongoing inflammation in the conjunctival epithelium and the recruitment and activation of neutrophils and lymphocytes. The overexpression of these immune mediators in scarring progressors after adjusting for C. trachomatis infection could reflect differences in exposure to other microbes or to irritants such as dust or smoke (23, 24). The strong association of *S100A7*, *IL8*, and *CXCL5* with TP emphasizes the importance of innate epithelial responses in driving pathological inflammation.

The association of scarring progression with *IDO1*, *SOCS3*, and *IL10*, all of which are regulators of inflammation, is likely a result of the host’s attempts to limit ongoing and pathological inflammation. While this may reflect that these individuals experienced greater inflammation than nonprogressors, it could also suggest that scarring progressors produce an excess of anti-inflammatory factors, leading to a poorer ability to clear infection. Genetic polymorphisms in interleukin 10 (IL-10) that lead to increased cytokine production and diminished cell-mediated immune responses to C. trachomatis have previously been reported (25, 26). However, in this scenario, one might expect the expression of these immune regulators to be increased in response to C. trachomatis infection in scarring progressors relative to that in nonprogressors, which was not the case.

*IL23A* and *IL19* were upregulated in individuals with scarring progression. *IL23A* was also upregulated in response to C. trachomatis infection in individuals with scarring progression and those prone to TP. IL-23A is a proinflammatory cytokine that is largely produced by dendritic cells and is essential for the survival and expansion of IL-17-producing Th17 cells (27). In this study, *IL17A* was strongly associated with TP but not with scarring progression. Furthermore, *IL21*, which was strongly upregulated in response to C. trachomatis infection (and to a lesser extent to TP), is an autocrine factor that sustains Th17 cells (28). Psoriasis is an autoimmune disease characterized by excessive cytokine production that is triggered by environmental stimuli on a background of genetic susceptibility (29). In psoriasis, keratinocytes recruit dendritic cells via CCL20, and the production of IL-23A by keratinocytes and dendritic cells recruits and activates IL-17A-producing Th17 cells, CD8^+^ T cells, innate lymphoid cells (ILC), and γδ T cells (30). IL-19 is produced by monocytes and epithelial cells and is also strongly upregulated in the keratinocytes of psoriasis patients (31, 32). IL-19 has been proposed as a member of the inflammatory IL-23A/IL-17A cascade in psoriasis; its upregulation in keratinocytes is driven by IL-17A, and it acts in an autocrine fashion on keratinocytes to amplify the effects of IL-17A (31). One such effect is the induction of S100A7 production, which contributes to sustaining the cycle of inflammation (33). In the intestine, IL-23A is thought to act synergistically with IL-1β to promote pathogenic ILC and Th17 responses following infection with Helicobacter hepaticus (34). IL-23A-responsive ILCs were also found to mediate intestinal pathology in a murine colitis model (35), and IL-1β-responsive IL-17A-producing ILCs have been reported in the conjunctiva (36). Together, these data suggest that the overexpression in scarring trachoma of *CCL20*, *IL23A*, *IL19*, *IL1B*, and *S100A7* could reflect mechanisms of pathogenesis similar to those observed in psoriasis and intestinal inflammation, whereby the upregulation of *IL23A* and *IL1B* promotes the recruitment and activity of pathogenic IL-23A-responsive cells (Th17, ILC, or γδ T cells, for example), which drive proinflammatory responses in the conjunctival epithelium, leading to chronic inflammation and fibrosis. The upregulation of *IL23A* in scarring progressors and TP-prone individuals in response to C. trachomatis infection could also suggest that greater polarization toward pathological IL-23A-responsive cell types could be involved in initiating and sustaining pathological proinflammatory cycles in the epithelium. Evidence from murine models suggests that chlamydial antigens can be maintained in distal tissues such as the gut for long periods of time (37), which could offer an additional explanation for prolonged inflammation through the continued stimulation of circulating cells.

*PDGFB* was upregulated in response to C. trachomatis infection in scarring progressors relative to nonprogressors; however, it was not independently associated with scarring progression. PDGFB is a key mediator of the wound-healing process; it can promote the recruitment and activity of neutrophils, fibroblasts, and macrophages, and it induces the production of matrix molecules from fibroblasts and induces fibroblast and myofibroblast contraction (38, 39). PDGFB could therefore directly contribute to scarring progression. *PDGFB* was upregulated in response to C. trachomatis infection and TP and was one of the genes most strongly associated with TP. The lack of direct association between scarring progression and *PDGFB* in a model adjusting for infection could indicate that its upregulation is tightly correlated with the presence of infection. The reason for its upregulation alongside *IL23A* in scarring progressors in response to C. trachomatis relative to expression in nonprogressors is unclear. A genome-wide association study of scarring trachoma suggested that host cell cycle, cell surface receptor signaling, and immune response pathways were associated with scarring, although no specific cytokine risk loci were identified (40).

The strengths of this study lie in the large sample size and the use of 11 longitudinal time points at which high-quality gene expression, infection, and clinical data are available. Of the genes associated with scarring progression, most had marginal fold changes of <1.4; however, small differences in multiple genes could act synergistically to favor pathological pathways (34). Although mucins *MUC7* and *MUC5AC* were strongly downregulated in response to infection, consistent with our results at baseline (4), we found little evidence in this study for dysregulation of mucins (hypothesized to lead to loss of epithelial barrier function) being associated with scarring progression. Supporting previous results from ourselves and others (4–6), *IFNG* was strongly upregulated in response to infection but was not associated with scarring progression (and was not strongly associated with TP), suggesting that a Th1-cell or NK-cell IFN-γ response is beneficial in the clearance of C. trachomatis infection. Upregulation of *IL21* and *IL17A* was strongly associated with infection and female sex, suggesting the involvement of Th17 cells in the antichlamydial immune response and that Th17 cell responses might be heightened in females relative to those in males. Sex differences in immune responses have previously been reported, including an increase in Th17 responses in the intestinal mucosa of females (41, 42). Given the potential role of the IL-23A/IL-17 axis in trachoma pathogenesis, this could offer a potential explanation as to why females are at greater risk of scarring trachoma than males (3). The majority of the genes tested were upregulated in younger participants, probably reflecting the decline in infection and disease incidence with increasing age. A caveat of this study is that gene expression data do not necessarily translate to changes in effector responses, and further research should aim to identify functional pathways of trachoma pathogenesis, including the phenotype and function of the cells responding to *IL23A*. We previously reported that mass azithromycin distribution (MDA) had an anti-inflammatory effect on conjunctival gene expression independent of the clearance of C. trachomatis infection, which was detectable by 3 (but not by 6) months posttreatment (10). However, analysis of the impact of MDA on gene expression in relation to scarring progression and whether it has a protective effect was outside the scope of this study.

### Conclusions.

Collectively, these data suggest that innate proinflammatory signals from the epithelium that drive leukocyte infiltration, IL-23A-responsive cells, and *SPARCL1*-, *MMP12*-, and *PDGFB*-mediated matrix reorganization and contraction are key pathways driving trachomatous scarring sequelae. The factors driving innate epithelial inflammation and causing scarring progressors to produce more *IL23A* and *PDGFB* in response to C. trachomatis infection remain unclear; they could have underlying genetic or epigenetic bases and/or be due to the presence of other organisms or irritants influencing the local immune response (43, 44). However, despite several studies, thus far there is limited evidence for any major infectious or genetic risk factors (40, 45). One could speculate that a complex combination of genetic or infectious risk factors increases an individual’s susceptibility, such that upon the trigger of C. trachomatis infection, individuals possessing these risk factors develop a bias toward pathological IL-23A-responsive cells, which lead to sustained proinflammatory responses in the conjunctival epithelium that are exacerbated by other stimuli. The concept of infectious triggers causing sustained inflammation and fibrosis has been illustrated in the intestinal epithelium, with a number of distinct molecular pathways (46). Further research should seek to verify these pathways of trachoma immunopathogenesis at the functional level. Nevertheless, *IL23A*, *SPARCL1*, and *PDGFB* may be key mediators driving pathological inflammation and fibrosis in trachoma, and molecules that inhibit their action could hold therapeutic potential in preventing scarring progression.

## MATERIALS AND METHODS

### Ethical approval.

This study was reviewed and approved by the Tanzanian National Institute for Medical Research, the Kilimanjaro Christian Medical Centre, and the London School of Hygiene & Tropical Medicine Ethics Committees and it adhered to the tenets of the Declaration of Helsinki. Written informed consent from a parent or legal guardian was requested from all study participants after detailed explanation in Swahili or Maa in the presence of a third person. A witnessed thumbprint was acceptable for consent if the individual was unable to read or write.

### Study design and trachoma control.

Study participants were recruited from three rural and predominantly Maasai villages in northern Tanzania. The study design and population have been described in detail in several earlier reports (4, 9, 10). In brief, a cohort of 616 children, aged 6 to 10 years at the beginning of the study in February 2012, were enrolled in the longitudinal cohort study and were visited every 3 months for 4 years, for a total of 17 time points.

The SAFE strategy (surgery for trichiasis [in-turned eyelashes], antibiotics, facial cleanliness, and environmental improvement) was implemented in the study villages by the field team in collaboration with district eye coordinators, following approval from the Tanzanian Ministry of Health (MoH). Education about environmental improvements and facial hygiene was provided by the field team, free trichiasis surgery was offered, and all members of the three villages (including study participants) were offered azithromycin for trachoma control during the August of the years 2012, 2013, and 2014.

### Clinical examination and sample collection.

At each time point, all available and consenting cohort participants were examined. Eyes were initially anaesthetized using preservative-free proxymetacaine hydrochloride 0.5% eyedrops. Each participant’s left eyelid was everted, and the tarsal conjunctiva was examined by an ophthalmic nurse experienced in trachoma grading using 2.5× loupes and a torch. Clinical signs were graded using the WHO detailed FPC grading system (47). Using the FPC grading system, F2/F3 corresponds to “trachomatous inflammation—follicular” (TF) and P3 corresponds to “trachomatous inflammation—intense” (TI) of the WHO simplified grading system (48). We consider P2 to also represent significant clinically apparent inflammation, and we therefore refer to P2/P3 as “TP” and use this designation in all analyses (2, 9, 10). At each time point, high-resolution conjunctival photographs were taken with a Nikon D90 camera with a 105-mm macro lens.

At baseline (time point 1, or time point 2 if not seen at time point 1) and final time points (time point 17, or time point 16 if not seen at time point 17), conjunctival photographs were independently graded by an ophthalmologist using a detailed scarring grading system (49). Baseline and final photographs for each individual were subsequently compared side by side in order to determine whether there was no progression (no scarring, or no progression of existing scarring) or incidence/progression of trachomatous scarring. Participant entry into the longitudinal study was permitted at time points 1 and 2, after which no new participants were enrolled.

At each time point swab samples were collected from the left tarsal conjunctiva using sterile polyester-tipped swabs (Puritan), as described previously (4). The first swab was collected into 250 μl RNAlater (Invitrogen), and the second was stored dry. Swabs were stored on ice in the field. RNAlater swabs were stored at 2 to 8°C overnight before transfer to −80°C for long-term storage. Dry swabs were stored immediately at −80°C.

### Chlamydia trachomatis detection.

At time point 1, DNA was extracted from dry-stored swabs using a PowerSoil DNA isolation kit (Mo Bio Laboratories), and this DNA was used for C. trachomatis detection by droplet digital PCR (ddPCR), as described previously (4). At time points 2 through 17, RNA and DNA were extracted from RNAlater-stored swabs using RNA/DNA purification kits (Norgen Biotek) following the manufacturer’s instructions, and this DNA was used for C. trachomatis detection by quantitative PCR (qPCR) (9, 10). Triplex qPCR was performed targeting chlamydial chromosomal (*omcB*) and plasmid (*pORF2*) targets and a human endogenous control gene (*RPP30*) (50). Samples were tested in duplicate and were determined positive if *RPP30* in combination with *pORF2* and/or *omcB* targets amplified for <40 cycles in one or both replicates. Norgen-extracted DNA from time point 2 was tested by qPCR and ddPCR for the C. trachomatis plasmid target for comparison, and the kappa score for agreement was 0.84 (10).

### Gene expression.

Norgen-extracted RNA from time points 1 to 5, 7, 9, 11, 13, 15, and 17 was reverse transcribed using SuperScript VILO cDNA synthesis kits (Thermo Fisher Scientific) following the manufacturer’s instructions. The relative abundances of 48 genes of interest, including those of *GAPDH* and *HPRT1* endogenous control genes, were quantified in each sample by qPCR using TaqMan microfluidic 384-well array cards (Thermo Fisher Scientific). qPCR was performed on a ViiA7 thermal cycler with TaqMan Universal mastermix, as described previously (4). The 46 genes of interest were shortlisted from a total of 91 genes tested at time point 1 (4), based on those most strongly associated with C. trachomatis infection and/or clinical signs. The original 91 genes were selected based on the results of previous cross-sectional studies and were centered around key biological processes hypothesized to underlie the immunopathogenesis of trachoma, including antimicrobial peptides, cell cycle regulators, cytokines and chemokines, biomarkers of epithelial-mesenchymal transition, matrix modifiers, the response to microbiota, mucins, NK cell markers, pattern recognition receptors, and signaling pathway regulators.

### Analysis.

Data were stored in Microsoft Access and were analyzed in STATA v15 and R (www.R-project.org). Gene expression data were normalized using the cycle threshold (Δ*C_T_*) method (51), normalizing the expression of each gene to the expression of *HPRT1* in the same sample. For quality control purposes, both genes and observations (defined as a sample from a participant at one time point) with >10% missing data were excluded. This resulted in the exclusion of three genes (*FGF2*, *SERPINB3-SERPINB4*, and *IL22*) and 61/4,853 observations, leaving 4,792 observations and 43 genes of interest (excluding *HPRT1* and *GAPDH*) in the analysis.

In order to assess the association between longitudinal gene expression and scarring progression, random effects linear regression models were performed for each gene in turn, using gene expression as the dependent variable and adjusting for infection, age, and sex, clustering on participant ID. These analyses were subsequently repeated for TP, adjusting for infection, age, and sex.

Random effects lasso regression models using the glmmLasso package in R (52) were used to select the genes most strongly associated with scarring progression and TP. This analysis does not permit any missing data, and therefore only complete cases were retained from the filtered data set described above. This resulted in a data set of 3,622 observations and 43 genes for the scarring progression analysis and a data set of 4,510 observations and 43 genes of interest for the TP analysis. Age, sex, and infection were adjusted for in each model.

In order to determine whether scarring progressors responded differently from nonprogressors to C. trachomatis infection at the gene expression level, the random effects linear regression analyses described above were repeated, including an interaction term between scarring progression status and C. trachomatis infection. These analyses were further repeated to investigate whether individuals prone to TP (defined as those who had TP at any time point) responded differently to infection relative to individuals in whom TP was never detected throughout the study duration. To provide an objective threshold for reporting the genes most strongly associated with the outcome, we highlight those genes with a *P* value below the level that controls the false discovery rate (FDR) to be less than 5%, using the Benjamini-Hochberg method (53).

### Data availability.

The longitudinal gene expression data set can be accessed on Figshare (https://doi.org/10.6084/m9.figshare.11401158.v1).

## Supplementary Material

Supplemental file 1
